# A Novel Loss-of-Sclerostin Function Mutation in a First Egyptian Family with Sclerosteosis

**DOI:** 10.1155/2015/517815

**Published:** 2015-04-23

**Authors:** Alaaeldin Fayez, Mona Aglan, Nora Esmaiel, Taher El Zanaty, Mohamed Abdel Kader, Mona El Ruby

**Affiliations:** ^1^Molecular Genetics and Enzymology Department, Human Genetics & Genome Research Division, National Research Centre, 33 El Bohouth Street (Former El Tahrir Street), P.O. Box 12622, Dokki, Giza, Egypt; ^2^Clinical Genetics Department, Human Genetics & Genome Research Division, National Research Centre, Egypt; ^3^Department of Medicine, Faculty of Medicine, Cairo University, Egypt; ^4^Department of Orodental Genetics, Medical Research Division, National Research Centre, Egypt

## Abstract

Sclerosteosis is a rare autosomal recessive condition characterized by increased bone density. Mutations in *SOST* gene coding for sclerostin are linked to sclerosteosis. Two Egyptian brothers with sclerosteosis and their apparently normal consanguineous parents were included in this study. Clinical evaluation and genomic sequencing of the *SOST* gene were performed followed by in silico analysis of the resulting variation. A novel homozygous frameshift mutation in the *SOST* gene, characterized as one nucleotide cytosine insertion that led to premature stop codon and loss of functional sclerostin, was identified in the two affected brothers. Their parents were heterozygous for the same mutation. To our knowledge this is the first Egyptian study of sclerosteosis and *SOST* gene causing mutation.

## 1. Introduction

Sclerosteosis (SOST1: MIM 269500) is an autosomal recessive sclerosing skeletal dysplasia in which bone overgrowth throughout life, affecting mainly the cranial and tubular bones, leads to distortion of facies and entrapment of cranial nerves. Syndactyly is a variable manifestation but represents an important diagnostic feature. The disorder is rare and the majority of affected individuals have been reported in the Afrikaner population of South Africa. A small number of individuals and families with sclerosteosis have been reported in other parts of the world, including Brazil, United States, Germany, Senegal, and Turkey [1].

Due to genetic heterogeneity, sclerosteosis was given 2 MIM numbers despite the same clinical features. Sclerosteosis-1 (SOST1: MIM 269500) is linked to a genetic defect in the* SOST* gene coding for sclerostin. The* SOST* gene product, sclerostin, is secreted by osteocytes and transported to the bone surface where it inhibits osteoblastic bone formation by antagonizing Wnt signaling [2]. Five different loss-of-function mutations relevant to sclerosteosis in* SOST* gene have been reported as pathogenic in ClinVar database to date [3]. Heterozygous and homozygous missense mutations in the* LRP4* gene were reported in 2 unrelated families with sclerosteosis-2 (SOST2: MIM 614305); also LRB4 and LRB5 interacted with sclerostin by Leupin et al. [4].

In this study, we sequenced the coding and exon-intron boundaries sequence of the entire* SOST* gene in two Egyptian brothers, offspring of consanguineous parents, with sclerosteosis. To our knowledge, this is the first Egyptian report of patients with sclerosteosis.

## 2. Subjects and Methods

This research complies with the standards established by the Medical Ethical Committee of the National Research Centre. A written informed consent was signed by the patients and their parents. The study included a consanguineous Egyptian family from Upper Egypt with 2 affected siblings with sclerosteosis.

### 2.1. Clinical Evaluation

The referred family was subjected to history taking, pedigree analysis, full clinical examination, and photography. Skeletal survey, hearing assessment, complete eye evaluation including fundus examination, CT scan, and MRI brain, abdominal ultrasound, complete blood picture, liver and kidney function tests, hormonal studies, and cytogenetic studies by G-banding using peripheral blood leukocytes were carried out for the 2 affected brothers.

### 2.2. Samples and DNA Extraction

After informed consent, three mL. of the peripheral blood samples was collected from the patients and their parents using K_2_EDTA as anticoagulant inside vacutainer sterile tubes. Genomic DNA was isolated from peripheral blood leukocytes by QIAamp DNAMini Kit (50 preps), catalog number 51304, Germany (https://www.qiagen.com/eg/). The parents denied molecular studies to the normal siblings.

### 2.3. Primers Designing

We designed the primers covering both two exons and exon-intron boundaries sequence using NCBI Primer-BLAST tool (http://www.ncbi.nlm.nih.gov/tools/primer-blast). Primers consist of three sets: one set covered whole exon 1 and exon 1-IVS1 boundary (from 17: 7109900 to 7110409) and two sets covered both IVS1-exon 2 boundary (from 17: 7107228 to 7107470) and last region of exon 2 (from 17: 7106785 to 7107253) as shown in Table 1.

### 2.4. Mutation Analysis

Mutation analysis of both exons and exon-intron boundaries sequence of the* SOST* gene was performed by conventional PCR using Alliance Bio Taq DNA Polymerase, catalog number M001TP20 (http://www.alliancebio.com/). Amplified fragments were verified by agarose gel electrophoresis containing ethidium bromide. The verified amplicons were purified and sequenced directly by ABI3730XL sequencer in Macrogen sequencing service, Korea [5].

### 2.5. In Silico Analysis

The sequencing data were analyzed by FinchTV version 1.4.0 [6]. Prediction of the disease causing mutations and putative effect of the mutations were identified using the Mutation Taster (MT) tool [7] and SNP annotation tool (SNPnexus) [8]. Exploration of the resulting protein was done by SWISS-MODEL tool [9] and UniProt database [10].

## 3. Results 

### 3.1. Clinical Data

**Figure 1:**
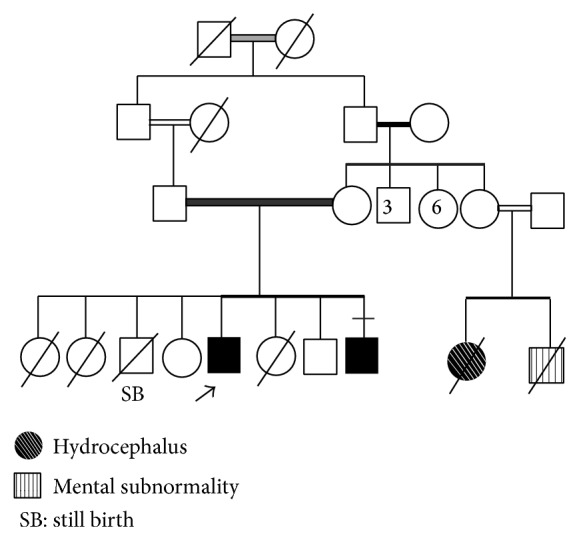
Family pedigree.

*Patient 1.* The proband is a 25-year-old Egyptian male who was referred to us because of progressive diminution of vision, hearing loss, and change in facial features during the last years. The patient is the offspring of first cousin parents. There was history of two older female siblings who died at the age of 4 months due to an unknown cause, a previous still birth at 7 months of gestational age, a younger female sibling who died at 20 years of age from atrial fibrillation, 2 normal female and male siblings, and a younger brother who began to show changes in facial features (patient 2). There was also family history of a maternal cousin who died at 4 years of age with hydrocephalus and his sibling with mental subnormality who died at 2 years of age; their parents are consanguineous. Family pedigree is shown in Figure 1. Pregnancy and delivery histories were uneventful.

At the age of 6 years, the parents noticed facial palsy and the proband's complaint of recurrent headache. A progressive course was reported with hearing affection and diminution of vision. By examination the patient was uncooperative and unable to communicate. He was tall (height was 183 cm = 2.0 SD above the mean) with increase in relative arm span (arm span was 191 cm) and he had a large head (head circumference 63 cm = 4.5 SD above the mean) and his weight was at the mean. Delayed milestones of development, delayed speech, and poor school performance were reported by the parents; however it was not possible to perform an intelligence quotient (IQ) test because of difficulty in hearing and vision and the incooperation of the patient.

**Figure 2:**
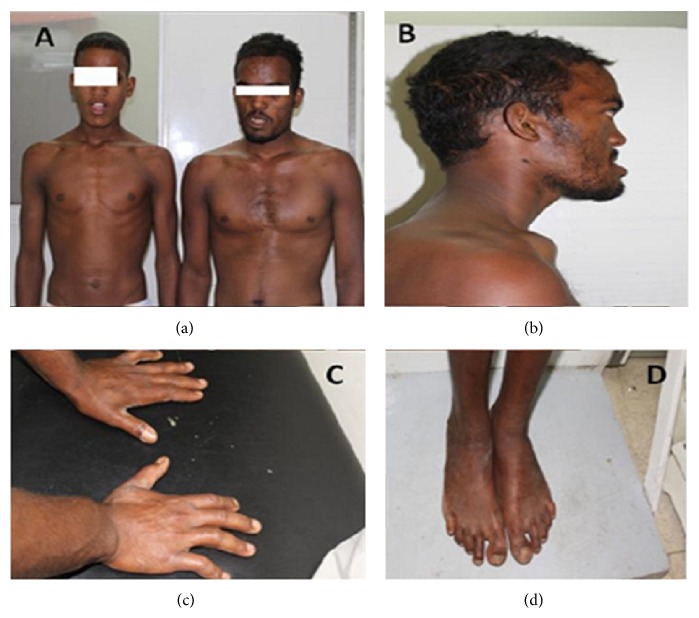
Proband (on the right hand side of the photo) and his affected brother (on the left hand side of the photo) showing distorted facies and strong body build (a); lateral view of the face of the proband showing proptosis and prognathism (b); both hands of the proband showing bilateral radial deviation of index fingers, camptodactyly of 5th fingers, and soft tissue syndactyly between fingers (c); both feet of the proband showing bilateral broad big toes with wide space between the 1st and 2nd toes, bilateral clinodactyly of 4th and 5th toes, and hypoplastic nails and soft tissue syndactyly between toes (d).

Facial features in the form of frontal bossing, high anterior hair line, prominent supraorbital ridges, proptosis, strabismus, depressed nasal root, midface hypoplasia, and prognathism were noted (Figures 2(a) and 2(b)). Orodental examination revealed receding premaxilla, macrognathia, thick alveolar ridge, high arched palate, malaligned irregular conical shaped teeth, and class II occlusion.

Skeletal examination revealed prominence of medial parts of the clavicles and upper ribs, bilateral radial deviation of index fingers, camptodactyly of 5th fingers, bilateral broad big toes with wide space between the 1st and 2nd toes, bilateral clinodactyly of 4th and 5th toes, and hypoplastic nails and soft tissue syndactyly between fingers and toes (Figures 2(c) and 2(d)). No history of previous bony fractures was reported and the patient denied further studies to assess bone density by DEXA scan.

**Figure 3:**
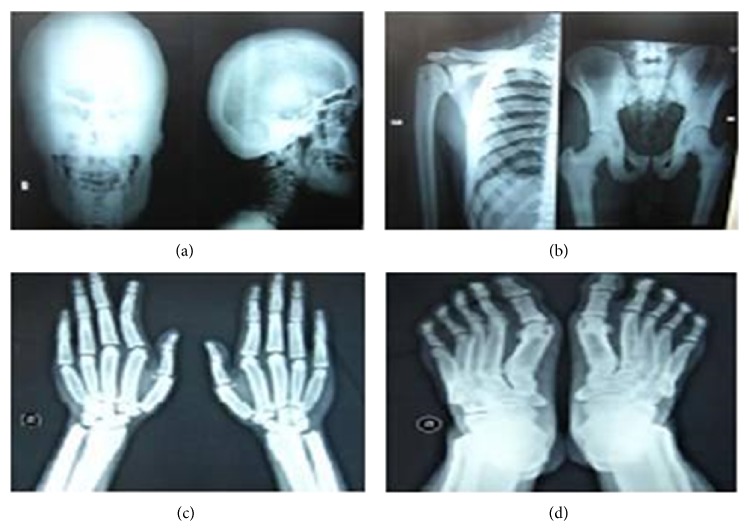
Radiological manifestations in patient 1. Skull X-ray (AP and lateral views) showing diffusely thickened dense calvarial bones of skull base and prognathism (a); X-ray chest (AP view) showing right clavicle and humerus and X-ray pelvis and upper part of both femora (AP view) with diffuse sclerotic texture and widened clavicles and ribs (b); X-ray of both hands (AP view) showing sclerotic appearance with medullary expansion of metacarpals and phalanges and hypoplastic terminal phalanges (c); X-ray of both feet (AP view) showing sclerotic appearance with medullary expansion of metatarsals and phalanges and hypoplastic terminal phalanges, bilateral hallux valgus, and overcrowded tarsal bones (d).

X-ray studies showed diffuse thick dense cranial bones, thick calvarium, narrow skull base foramina, normal size and shape of sella turcica, hypoplastic maxilla, protruded mandible, diffuse sclerotic texture of long bones with medullary cavity expansion of metacarpals, and metatarsals and phalanges of hands and feet (Figure 3). MRI of the brain denoted no pituitary gland lesions with patent cavernous sinuses and inferior sagging of the infundibular recess of third ventricle.

Complete blood picture, coagulation profile, liver function tests, and kidney function tests were normal. Growth hormone level and thyroid profile were normal. Audiogram showed bilateral profound mixed hearing loss. Eye evaluation and fundus examination revealed hypotropia, exotropia, and bilateral optic atrophy. Abdominopelvic ultrasound was normal. Cytogenetic analysis by G-banding using peripheral blood leukocytes denoted normal male karyotype in all studied metaphases (46, XY).

*Patient 2.* He was a 17-year-old male patient, the younger brother of patient 1. He began to show changes of facial features at the age of 15 years. By examination he was cooperative and tall (height: 188 cm = 3.0 SD above the mean) with increase in relative arm span (arm span: 194 cm) and he had a large head (head circumference 60 cm = 3.0 SD above the mean) and his weight was at the mean. He had long face, broad forehead, depressed nasal root, midface hypoplasia, short philtrum, and macrognathia (Figure 2(a)). Open mouth with fissured lower lip, thick alveolar ridge, macroglossia with median grooved tongue, high arched palate, torus palatines, short uvula, and enamel hypocalcification were detected by orodental examination. Mild deafness was noted. Broad chest with medial prominence of clavicles and long neck were other features (Figure 2(a)). Examination of limbs revealed bilateral camptodactyly of 2nd, 3rd, and 5th fingers, broad interphalangeal joints, partial syndactyly between 2nd, 3rd, 4th, and 5th right fingers, and complete syndactyly between the left 2nd and 3rd fingers; operated upon, bulbous big toes with wide space between 1st and 2nd toes and clinodactyly of 4th and 5th toes bilaterally. No history of previous bony fractures was reported and the patient and his parents denied further studies to assess bone density by DEXA scan.

### 3.2. Molecular Analysis

#### 3.2.1. Sequencing Results

**Figure 4:**
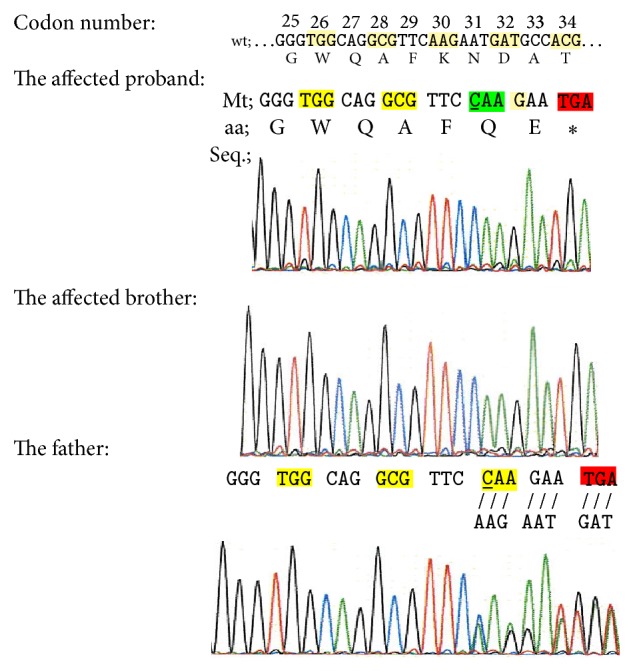
Paternal partial sequence chromatogram displaying the DNA sequence of affected proband, brother, and father (the mother showed the same father sequence chromatogram). The lined nucleotide indicates the position of the homozygous one nucleotide insertion (c.87_88insC, NCBI_rs377648601) resulting in a frameshift mutation and early stop codon. (∗) and red box indicate the position of TGA stop codon. (/) indicates the substituent nucleotides. wt: wild type; Mt: mutant type; and aa: amino acids.

Direct sequencing of the* SOST* gene data, in the studied family, was analyzed by FinchTV software and revealed a novel homozygous c.87_88insC mutation (it was registered by the author under NCBI_rs377648601) in exon 1 for the proband and his brother (nucleotide numbering refers to the cDNA RefSeq using the A of the ATG translation initiation codon as nucleotide +1). This nucleotide change was confirmed in his parents in the heterozygous state (Figure 4). This mutation is not recorded in the NCBI (dbSNP), Ensembl SNPs, 1000 Genomes Project (TGB), the Human Gene Mutation Database (public HGMD), ClinVar, The Exome Aggregation Consortium (ExAC) browser, and NHLBI Exome Sequencing Project (ESP) server databases.

#### 3.2.2. Effect Prediction of c.90_91insC Mutation on Sclerostin Function

**Figure 5:**
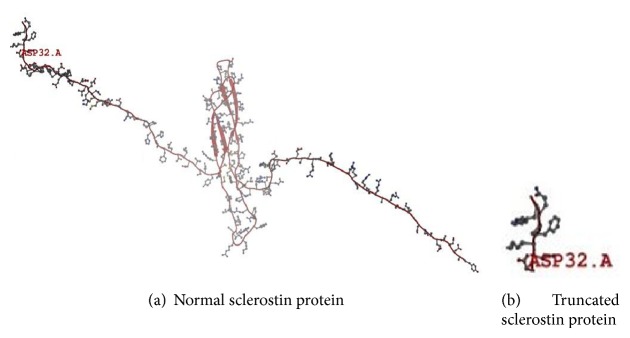
Sclerostin model for wild type (a) and mutant sclerostin protein (b) where sclerostin protein was truncated at Asparagine (ASP) amino acid at codon 32 to present premature stop codon (PTC) D32X resulting in the discovered c.87_88insC mutation.

**Figure 6:**
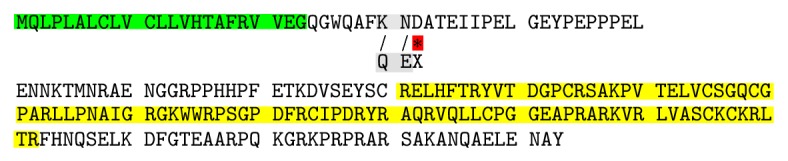
Sclerostin protein sequence illustrates the first 23 putative secretory signal sequences highlighted in green and CTCK domain highlighted in yellow. Altered amino acids are shadowed with gray highlighted substituents, and red star (∗) indicated to end of the truncated sclerostin protein.

Using SNP annotation tool (SNPnexus) (http://snp-nexus.org/) to predict possible function of c.87_88insC mutation, we found that this mutation led to K30Q substitution and frameshift stop-gain resulting in acquired early stop codon (TGA) at codon 32 producing truncated nonfunctional sclerostin protein. To explore the truncated protein resulting in this mutation, we used SWISS-MODEL tool and UniProt database, where it was found that this mutation led to the production of 8-oligopeptide chain only after removing the first 23 signal peptide amino acids; besides that the resulting truncated protein did not contain C-terminal cysteine knot-like (CTCK) domain (Figures 5 and 6).

#### 3.2.3. Prediction Disease Causing

By using Mutation Taster (MT) tool to predict the extent to which c.87 88insC mutation may cause the occurrence of disease, we found that this mutation led to amino acid change K30Q with further changes downstream to create premature stop codon (PTC) D32X (Figure 6). Depending on bioinformatics prediction tools which were used in this study, we predicted that this mutation led to shortened truncated sclerostin (8 oligopeptides only) and created PTC at a 32 residue. Hence, because of D32X located away about 125 upstream nucleotides from the first exon-exon junction complex, the mutant mRNA will be predicted to degrade by NMD pathway to prevent it to translate; this relies on the reported reference stating that PTC which is located further than about 50 nucleotides upstream of the exon-exon junction complex triggers NMD. So, this mutation has a pathogenic effect and is regarded as a disease mutation (full data shown in the Appendix).

## 4. Discussion

Sclerosteosis is a severe sclerosing skeletal genetic disorder. The disease affects bone modeling and remodeling, especially in the skull and diaphyseal region of long bones. The increased rate of bone formation suggests a defect in osteoblast function [11]. Although the condition is rare, the vast majority are present in the Afrikaner population of Dutch descent in South Africa. A small number of individuals and families with sclerosteosis have been reported in other parts of the world, including Brazil, United States, Germany, Senegal, and Turkey [1]. The rarity of sclerosteosis led to knowledge gaps in the molecular and cellular basis of this disease and resulted in unavailable satisfactory diagnostic and therapeutic strategies.

In this study, two Egyptian patients, offspring of consanguineous parents, were reported. The progressive bone overgrowth leading to tall stature, distorted facies, frontal prominence with midface hypoplasia, mandibular prognathism, dental malocclusion, and progressive bony encroachment upon the middle ear cavities and auditory nerve canals causing deafness in addition to optic nerve atrophy was consistent with sclerosteosis.

Sclerosteosis must be differentiated from osteopetrosis and other sclerosing bone dysplasias [12]. Autosomal recessive van Buchem disease (VBCH: MIM 239100) is clinically similar to sclerosteosis. However, the main clinical differences between the 2 diseases are gigantism and hand abnormalities present in sclerosteosis but never in VBCH. In our studied sibs, the tall stature, skeletal overgrowth, presence of syndactyly, irregular teeth with malocclusion, torus palatines, and nail hypoplasia support the diagnosis of sclerosteosis.

Balemans et al. [13] assigned the locus for sclerosteosis to 17q12–q21, the same general region as the locus for VBCH because of the clinical similarities. However, the possibility that sclerosteosis and VBCH are caused by mutation in the same gene appeared to have been excluded by the study of Brunkow et al. [14], in which mutations in the coding region of the* SOST* gene were found in cases of sclerosteosis but not in cases of VBCH. Further studies by Van Hul et al. [15] and Balemans et al. [16] revealed that VBCH was caused by a 52-kb deletion approximately 35 kb downstream of the* SOST* gene that removes a* SOST*-specific regulatory element. Loots et al. [17] concluded that VBCH is caused by deletion of a* SOST*-specific regulatory element and is allelic to sclerosteosis.

**Figure 7:**
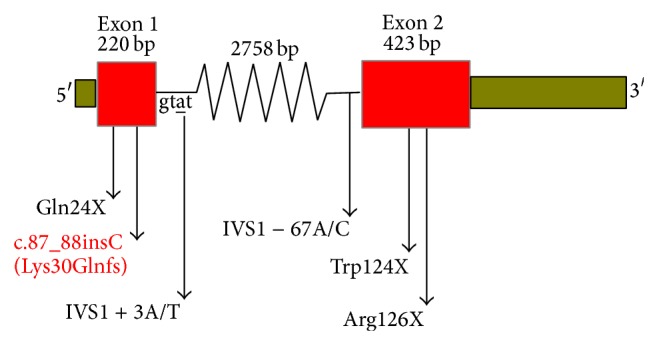
Schematic representation of sclerosteosis relevant reported ClinVar pathogenic mutations in* SOST* gene. The new mutations are shown in red color.

Mutations in human* SOST* are responsible for sclerosteosis-1.* SOST* is a negative regulator of bone formation [2]. According to ClinVar database, there are five* SOST* gene mutations that have pathogenic effect causing sclerosteosis. The current study is the first to detect 1 bp cytosine insertion in codon number 30 (c.87_88insC) as frameshift mutation resulting in early stop codon at number 32, and by using bioinformatics prediction tools, it likely to be pathogenic mutation to give nonfunctional short sclerostin protein (Figure 7).

In silico analysis of the discovered new frameshift stop gained mutation revealed that it is a pathogenic mutation that led to nonfunctional sclerostin protein. This is supported with what was mentioned in patients with sclerosteosis in which premature termination codons in the* SOST* gene lead to an inhibitory effect of the gene product sclerostin on bone formation that was supported by the inhibited proliferation and differentiation of mouse and human osteoblastic cells after the addition of exogenous sclerostin to osteogenic cultures [18–20].

The clinical manifestations reported in the studied brothers in the form of tall stature, distorted facies, orodental findings, syndactyly, and encroachment of cranial nerves are consistent with sclerosteosis and mutation in* SOST* gene. However, the relative increase of arm span compared to height was not previously noted in the literature. Also, of note, the presence of learning difficulties in patient 1 in this study has not been reported before in sclerosteosis. Although this can be an expansion of the phenotypic characteristics of the syndrome, the maternal history of intrauterine fetal death, unexplained infant deaths, and other affected family members with mental subnormality and hydrocephalus strongly suggest the possibility of another inherited disorder in this consanguineous family, which calls for further studies in this family.

## 5. Conclusions

Up to our knowledge, this is the first study of Egyptian patients with sclerosteosis describing the associated clinical traits and sclerosteosis causing mutations in* SOST* gene. We identified a novel mutation in* SOST* gene, in two affected Egyptian brothers with sclerosteosis, characterized by one nucleotide insertion resulting in a frameshift mutation. In silico analysis indicated that this nucleotide sequence change, classified as severe disruptive mutation, resulted in loss-of-sclerostin function. The clinical traits are compatible with the genetic investigations except for learning difficulties in patient 1 that require further studies to identify their genetic background.

## Figures and Tables

**Table 1:** Sequence of designed PCR primers for* SOST* gene.

Name	Forward (5′-3′)	Reverse (5′-3′)	Ta^*^
SOST1	CTGAGGGAAACATGGGACCAGC	CAGGCAAGACTGTTCCTCGACCA	56
SOST2	ACAGGGTGCGCAGGAGAGCTT	CCCATCGGTCACGTAGCGGG	56
SOST3	ACTTCACCCGCTACGTGACCGA	CACGCGCAGAGGACAGAAATGT	55

^*^Ta means “Annealing Temperature.”

**Table 2:** Data of Mutation Taster (MT) tool about prediction of effect of c.87_88insC mutation.

Summary	□ NMD
	□ Amino acid sequence changed
	□ Frameshift
	□ Protein features (might be) affected
	□ Splice site changes
Analysed issue	Analysis result

Name of alteration (HGNC)	*SOST *
Alteration (phys. location)	chr17: 41836022_41836023insG
Alteration type/region	Insertion/CDS
AA changes	K30Qfs^∗^3
Position(s) of altered AA	30 (frameshift or PTC further changes downstream)
Frameshift	Yes
phyloP/phastCons	2.271, 1 (flanking)/0.445, 1 (flanking)
Length of protein	NMD
Position (AA) of stop codon in wt/mu AA sequence	214/32
Theoretical NMD boundary in CDS	170
Wild type AA sequence	MQLPLALCLVCLLVHTAFRVVEGQGWQAFKNDATEIIPE LGEYPEPPPELENNKTMNRAENGGRPPHHPFETKDVSEY SCRELHFTRYVTDGPCRSAKPVTELVCSGQCGPARLLPN AIGRGKWWRPSGPDFRCIPDRYRAQRVQLLCPGGEAPRA RKVRLVASCKCKRLTRFHNQSELKDFGTEAARPQKGRK PRPRAR SAKANQAELE NAY^∗^
Mutated AA sequence	MQLPLALCLVCLLVHTAFRVVEGQGWQAFQ E^∗^

All positions are in base pairs (bp) if not explicitly stated differently. AA/aa: amino acid; CDS: coding sequence; mu: mutated; NMD: nonsense-mediated mRNA decay; nt: nucleotide; wt: wild type; TGP: 1000 Genomes Project.

## Appendix

See Table 2.
